# Vancomycin disrupts mitochondrial morphology and function and impairs macrophage fungal killing

**DOI:** 10.1128/mbio.00580-26

**Published:** 2026-04-29

**Authors:** Ebrima Bojang, Lozan Sheriff, Emma Morris, Sophie Rouvray, Man Shun Fu, Chloe Wellings, Ketema Abdissa, Victoria Stavrou, Callum Clark, Andrew D. Southam, Warwick B. Dunn, David Bending, Jose R. Hombrebueno, Ilse Jacobsen, Sarah Dimeloe, Rebecca A. Hall, Rebecca A. Drummond

**Affiliations:** 1 Institute of Immunology & Immunotherapy, University of Birmingham 1724, Birmingham, United Kingdom; 2 Department of Genetics and Genome Biology, University of Leicester 4488, Leicester, United Kingdom; 3 Research Group Microbial Immunology, Leibniz Institute for Natural Product Research and Infection Biology-Hans Knoell Institute, Jena, Germany; 4 Institute of Microbiology & Infection, University of Birmingham 1724, Birmingham, United Kingdom; 5 Phenome Centre Birmingham and School of Biosciences, University of Birmingham 1724, Birmingham, United Kingdom; 6 Centre for Metabolomics Research, Department of Biochemistry, Cell and Systems Biology, Institute of Systems, Molecular and Integrative Biology, University of Liverpool 4591, Liverpool, United Kingdom; 7 Institute of Inflammation and Ageing, University of Birmingham 1724, Birmingham, United Kingdom; 8 Institute of Microbiology, Friedrich Schiller University Jena 9378, Jena, Germany; 9 Kent Fungal Group, University of Kent 2240, Canterbury, Kent, United Kingdom; Hebrew University of Jerusalem Robert H. Smith Faculty of Agriculture Food and Environment, Rehovot, Israel

**Keywords:** *Candida albicans*, mitochondria, macrophages, hospital infections, postantibiotic effect, vancomycin, fungi

## Abstract

Vancomycin is a widely prescribed antibiotic used in the treatment of gram-positive bacterial infections. We previously showed that this antibiotic disrupted protective antifungal immune responses via microbiome dysbiosis, enhancing susceptibility to invasive candidiasis. Antibiotics are an independent risk factor for developing this life-threatening fungal infection, but whether microbiota-independent mechanisms also drive this association is not clear. Here, we show that vancomycin directly impairs macrophage responses to *Candida albicans*, the main causative agent of invasive candidiasis. Vancomycin-treated macrophages were less able to kill *C. albicans* despite normal phagocytosis rates and were hyper-inflammatory and more likely to die during infection. Using a fluorescently labeled vancomycin, we observed vancomycin uptake by macrophages *in vivo* and within close proximity to the mitochondrial outer membrane. Vancomycin treatment led to a significant depolarization, reduced respiratory capacity, and a hyper-fragmented morphology of mitochondria, as well as increased cellular ROS production. Taken together, this work demonstrates direct effects of vancomycin on mammalian immune cells, helping us to understand the pro-inflammatory effects of this drug and how it promotes susceptibility to life-threatening fungal infection.

**IMPORTANCE:**

Antibiotics are widely prescribed drugs used to treat bacterial infections; however, their use may increase the likelihood of developing life-threatening fungal infections in vulnerable patients. *Candida albicans* is a commensal fungus in humans but may cause serious disease in patients with defined risk factors, including antibiotic exposure. We find that the antibiotic vancomycin significantly impairs the ability of macrophages to kill *C. albicans* yeast. Vancomycin-induced defects in fungal killing were associated with changes to mitochondria in antibiotic-exposed macrophages, which also exhibited enhanced oxidative stress and reduced survival during fungal infection. This work identifies a direct mechanism by which antibiotics may impair antifungal immunity.

## INTRODUCTION

Antibiotics are an independent risk factor for developing mucosal and systemic infections with *Candida* fungi, most commonly *Candida albicans* (1). Antibiotics deplete commensal bacteria that may provide protection against these fungal infections. For example, *Lactobacillus* species prevent inflammatory growth of *C. albicans* in the vaginal mucosa, and the depletion of these bacteria may promote the development of vaginitis and recurring *C. albicans* infections (2, 3). In the gut, commensal *C. albicans* populations are relatively low in abundance in most people but can significantly increase following treatment with antibiotics (4, 5). These “fungal blooms” have been observed in patients prior to development of an invasive *C. albicans* infection (6), indicating that outgrowth of *C. albicans* in the gut is a significant risk factor for life-threatening systemic infection.

We previously published that antibiotics also impair antifungal immune responses in an organ-specific manner, leading to the development of fungal and bacterial co-infection and increased mortality in the context of antibiotic treatment (7). Immunocompetent mice pre-treated with broad-spectrum antibiotics, but particularly vancomycin, had disrupted Th17 responses in the intestines due to the depletion of segmented filamentous bacteria, which resulted in increased fungal burdens in the GI tract and escape of commensal bacteria to the periphery (7). In that work, antibiotics appeared to be mediating immune system defects indirectly via changes to the microbiota. However, some antibiotics can directly impair mammalian cell function. For example, bactericidal antibiotics, including quinolones and β-lactams, were shown to significantly impair mitochondrial respiration and cause oxidative stress within mammalian epithelial cells (8). Some antibiotics have been shown to interfere with the phagocytic function of macrophages (9, 10), but whether antibiotics directly impair antifungal functions of these cells is not known. Given the critical role macrophages play in the innate immune response to *C. albicans* infections (11), we examined how antibiotic treatment affected macrophage uptake and killing of *C. albicans*. We chose to focus on vancomycin since we previously showed that single treatment with this antibiotic enhances mortality following intravenous challenge with *C. albicans* (7). We found that vancomycin impaired *C. albicans* killing by macrophages without affecting phagocytosis, instead causing mitochondrial defects and an enhanced inflammatory phenotype.

## RESULTS

### Vancomycin impairs fungal killing by macrophages

**Fig 1:**
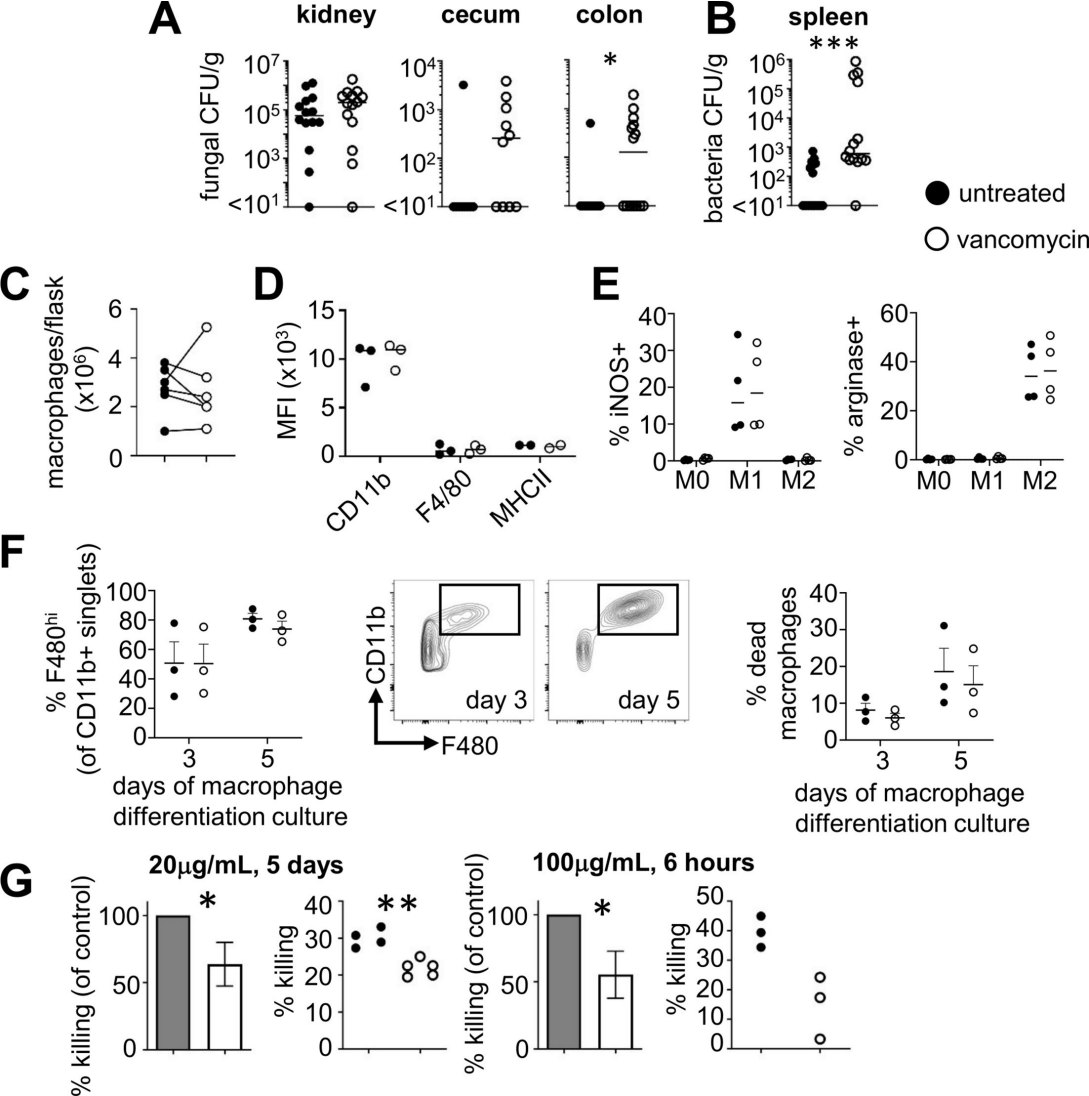
Vancomycin directly impairs fungal killing by macrophages. (**A**) Wild-type C57BL/6 mice were treated with vancomycin in the drinking water for 4 weeks, or left untreated, prior to intravenous infection with *C. albicans* SC5314. Indicated organs were isolated at day 7 post-infection for fungal or (**B**) bacterial burden analysis. Each point represents an individual animal. Data pooled from three independent experiments and analyzed by Mann-Whitney *U*-test. (**C**) The number of macrophages cultured per flask from mouse bone marrow in the presence or absence of vancomycin. Lines show paired samples that used bone marrow cells from the same animal for macrophage differentiation. (**D**) Mean fluorescence intensity (MFI) of indicated lineage and activation markers on macrophages cultured without antibiotics (untreated) or 20 µg/mL vancomycin during differentiation. Each point represents macrophage cultures generated independently from different animals, and the line marks the mean value. (**E**) The frequency of macrophages staining positive for iNOS or arginase (by flow cytometry) after being cultured in M1 (LPS and IFNγ) or M2 (IL-4) polarization conditions, compared to unstimulated (M0). Each dot represents macrophages generated from an individual animal, analyzed on different days, and the line marks the mean value. (**F**) The frequency of F4/80^hi^ macrophages (within CD11b^+^ singlets gate, left) and the frequency of dead macrophages (ZombieViolet^+^, right) at days 3 and 5 post-setup of the macrophage differentiation culture, with example FACS plot showing gating for F480^hi^ cells. Each dot represents macrophages generated from an individual animal, analyzed on different days. Data shown as mean ± SEM. (**G**) Fungal killing ability of macrophages that were either exposed to vancomycin during differentiation from bone marrow (i.e., 5 days) (left) or treated with vancomycin for 6 h after differentiation had been completed (right). Bar graphs represent means from two to three pooled independent experiments, shown as mean ± SEM (analyzed by Mann-Whitney *U*-test); dot plots show technical replicates from an example experiment (analyzed by unpaired *t*-tests). *P < 0.05, **P < 0.01, ***P < 0.005.

We previously found that vancomycin disrupted control of fungal infection in the GI tract and increased intestinal permeability, leading to escape of commensal bacteria, which was associated with increased mortality (7). In an independent animal facility, we confirmed that vancomycin-treated mice had increased fungal burdens specifically within the GI tract following intravenous challenge with *C. albicans* (Fig. 1A). Moreover, we observed increased burdens of bacteria in the spleens of vancomycin-treated mice (Fig. 1B), in line with our previous observations (7). We previously linked this phenotype with antibiotic-driven dysbiosis that led to disruption of lymphocyte responses within the GI tract, including reduced production of GM-CSF and IL-17 (7). These cytokines act on myeloid cells to activate phagocytosis and antifungal killing pathways (11). However, antibiotics may also directly affect mammalian cell responses in the absence of a microbiota or lymphocytes (10). To determine whether vancomycin has a direct effect on myeloid cells, we moved to an *in vitro* system to test the effects of the antibiotic without confounding factors caused by microbiota dysbiosis and changes in lymphocyte functional phenotype. We differentiated mouse bone marrow-derived macrophages in the presence of vancomycin or in antibiotic-free media and examined macrophage maturation and viability. We found that differentiating macrophages in the presence of vancomycin had no effect on the number of macrophages generated, the expression of activation/maturation markers, or ability to functionally polarize after 5 days of differentiation (Fig. 1C through E). We also observed no difference in the frequency of mature F480^hi^ macrophages at an early time point during differentiation (day 3), and viability of the macrophages remained similar between conditions at both time points tested (Fig. 1F). Next, we challenged the macrophages with live *C. albicans* and measured their fungal killing capacity by comparing yeast cell viability after incubation with the macrophages. We found that vancomycin-treated macrophages had significantly impaired fungal killing, regardless of whether vancomycin treatment took place during differentiation (20 µg/mL for 5 days) or occurred post-differentiation (100 µg/mL for 6 h) (Fig. 1G). These data demonstrate that vancomycin impairs macrophage killing of *C. albicans*.

### Vancomycin does not impair uptake of live *C. albicans* by macrophages

Antibiotics have been previously shown to disrupt phagocytosis (9, 10), and vancomycin has been described to block autophagy pathways that are involved in uptake of fungi and bacteria (12). We therefore explored whether vancomycin disrupted phagocytosis of macrophages, leading to the impaired killing we observed. For all these and subsequent experiments, we used the long-term exposure to the lower dose of vancomycin (20 µg/mL), since this dose was previously measured in vancomycin-treated humans (13).

**Fig 2:**
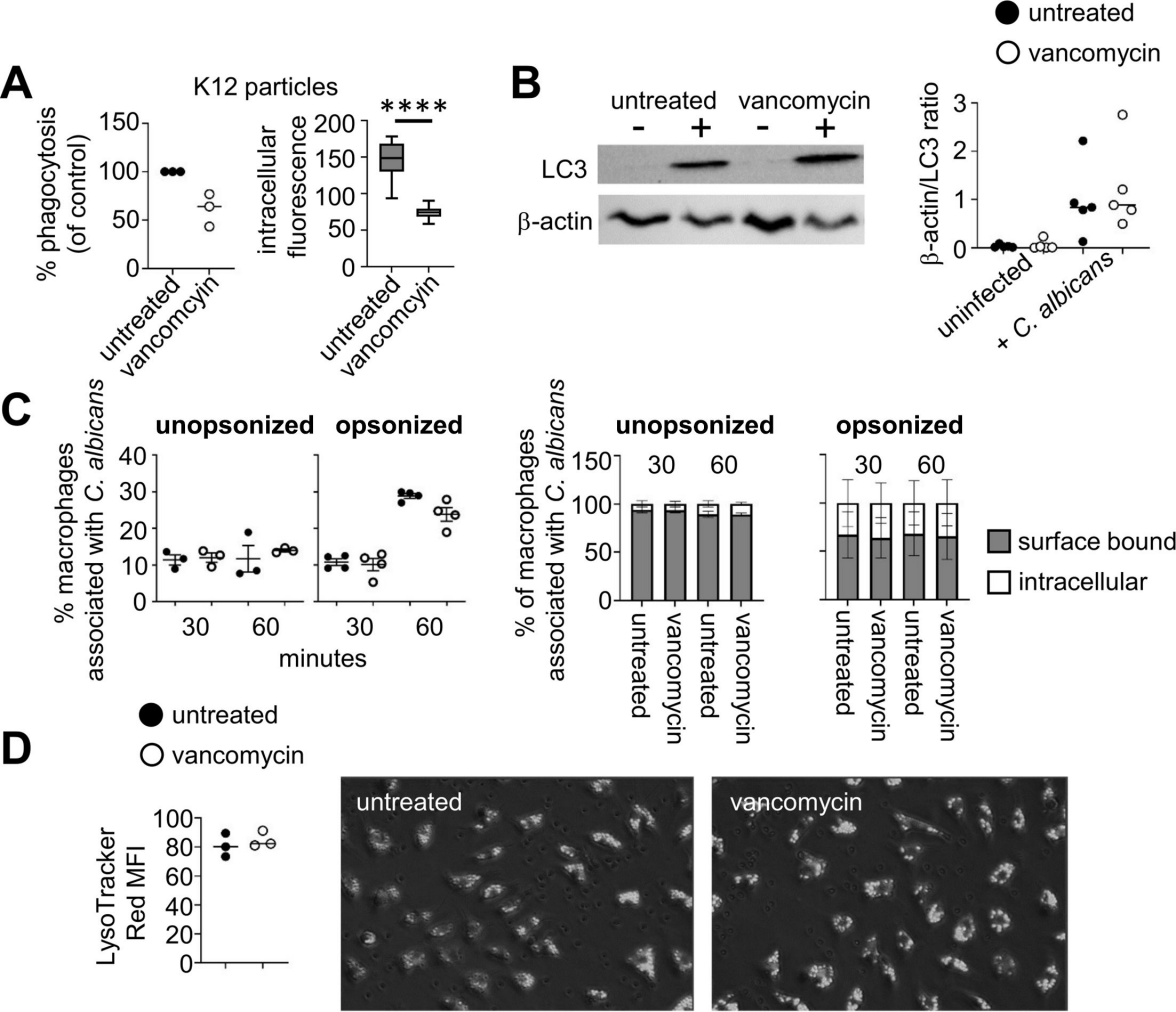
Vancomycin does not affect phagocytosis of *Candida albicans*. (**A**) Phagocytosis assay with fluorescent K12 *Escherichia coli* particles. Dot plot shows the mean value of technical replicates from individual experiments, where each point represents macrophages generated from different animals. Line represents mean value. Box-and-whisker plot (showing min-to-max) shows representative data from one of these experiments (*n* = 10 technical replicates per condition) and was analyzed by unpaired *t*-test. ****P < 0.0001. (**B**) Example Western blot for LC3 and β-actin from macrophages that were uninfected (−) or infected with live *C. albicans* for 2 h (+). The graph shows quantification of pixel densities expressed as a ratio between the two proteins. Each point represents an individual experiment (*n* = 5 total, line represents the mean). (**C**) Phagocytosis assay (by flow cytometry) with live *C. albicans* that was either unopsonized or opsonized with live mouse serum. Each point represents the mean value of technical replicates from individual experiments using macrophages generated from individual animals. Data shown as mean ± SEM. Bar graphs show mean values from individual experiments, shown as mean ± SEM. (**D**) Quantification of mean fluorescence of LysoTracker Red staining in macrophages challenged with heat-killed *C. albicans* at 30 min post-stimulation. Each point represents an average of 5-6 fields of view from three independent experiments, and the line represents the mean. Example images are from 30 min post-stimulation.

First, we measured general phagocytic capacity by challenging untreated and vancomycin-treated macrophages with fluorescent particles, which are made from bacterial extract and largely recognized by macrophages via TLR4. This experiment revealed that vancomycin pre-treatment impaired macrophage phagocytosis of these particles (Fig. 2A). To examine potential underlying reasons for this, we measured the abundance of the autophagy protein LC3 in macrophages using Western blot. LC3-associated phagocytosis (LAP) is a process involving components of autophagy machinery that enable uptake of microbes and activation of inflammatory responses, including to *C. albicans,* since LC3-deficient macrophages are impaired in their ability to kill yeast cells (14). Vancomycin was previously indicated to disrupt LC3 abundance and autophagy pathways (12); hence, we examined whether similar disruptions occurred in our model. We found that infection of macrophages with *C. albicans* significantly upregulated LC3 abundance, but vancomycin did not alter this across several experiments (Fig. 2B). Next, we examined whether vancomycin pre-treatment specifically affected *C. albicans* uptake, which is predominantly driven by β-glucan receptors (such as dectin-1 and CR3). For that, we performed a flow cytometry-based phagocytosis assay in which macrophages are challenged with fluorescent *C. albicans*, and surface-bound and internalized yeast cells were distinguished using a counter-stain with anti-*Candida* antibody. In contrast to our observations with fluorescent particles, we observed no significant difference between untreated and vancomycin-treated macrophages in their uptake or binding of live *C. albicans* (Fig. 2C). Taken together, these experiments show that vancomycin treatment may generally impair macrophage phagocytosis, but these defects are less relevant for uptake of live *C. albicans,* and reduced phagocytosis does not explain the killing defects of these cells.

### Phagosome maturation is not affected by vancomycin treatment

Following phagocytosis of live *C. albicans*, macrophages must initiate pathways to enable maturation of fungus-containing phagosomes. This includes recruitment of proteins such as Rab GTPases and a reduction in the pH of the phagosomal lumen, which contributes toward pathogen killing and prevents *C. albicans* from forming hyphae that enable escape from the macrophage (15). We examined phagosome maturation in untreated and vancomycin-treated macrophages using the pH-sensitive dye Lysotracker Red and live cell imaging to observe the development of an acidic environment within fungus-containing phagosomes (Fig. 2D). For these experiments, we used heat-killed *C. albicans,* since live fungal cells have been shown to neutralize phagosome pH (16). These experiments revealed no significant impairment of phagosomal acidification by vancomycin-treated macrophages (Fig. 2D). Therefore, vancomycin does not impair uptake of *C. albicans* nor prevent the acidification of fungus-containing vacuoles.

### Vancomycin does not directly affect *Candida albicans* growth or hyphae induction

Since vancomycin-treated macrophages had impaired *C. albicans* killing yet similar phagocytosis rates and phagosome maturation, we considered whether vancomycin could have a direct impact on *C. albicans* and inhibit fungal growth, giving rise to what appeared to be a killing defect in our macrophage co-culture assays. To examine this possibility, we incubated vancomycin with *C. albicans* directly and measured the impact on growth, hyphal formation, and exposure of chitin in the cell wall. We found that vancomycin had no impact on *C. albicans* growth or on the formation of hyphae (Fig. S1A and B). Exposure of chitin on the cell wall was also not affected by vancomycin treatment (Fig. S1C). Therefore, vancomycin does not significantly affect *C. albicans* directly.

### Vancomycin-treated macrophages are more likely to die following *C. albicans* infection

**Fig 3:**
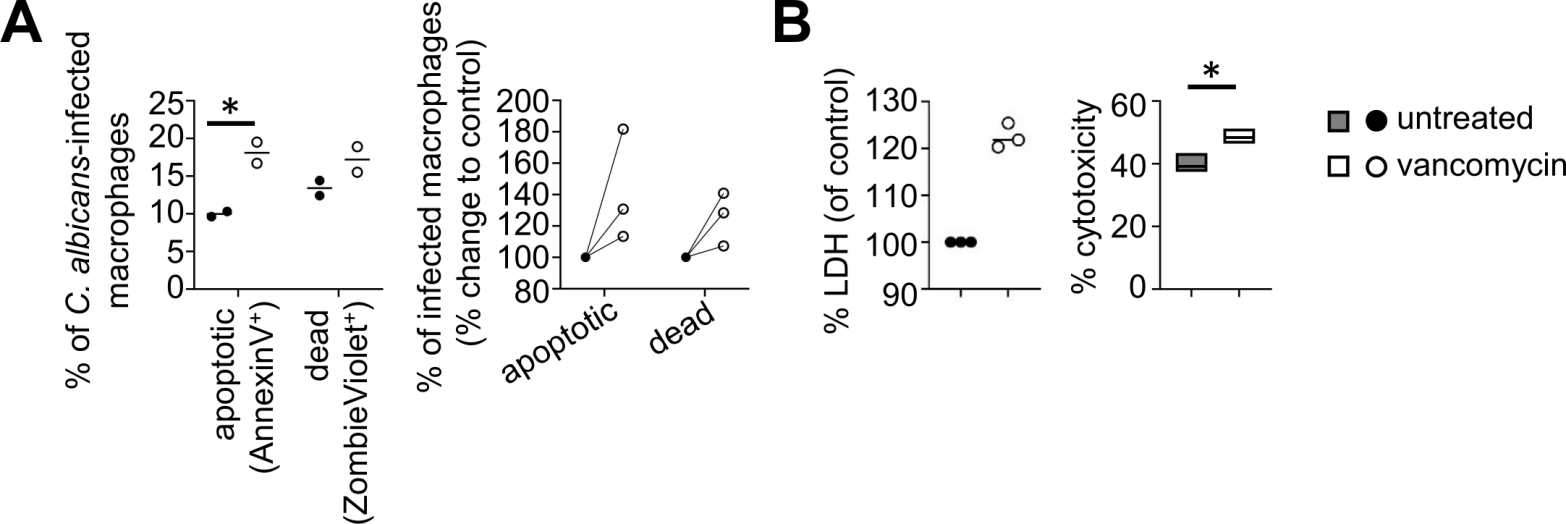
Vancomycin-treated macrophages are more prone to cell death following *C. albicans* infection. (**A**) Frequency of apoptotic (annexin-V^+^ZombieViolet^−^) and dead (annexin-V^+^ZombieViolet^+^) macrophages that had bound or internalized *C. albicans*. Left graph shows two technical replicates in a single experiment (line represents the mean), analyzed by two-way ANOVA. *P < 0.05. The right graph shows pooled data using macrophages generated from different animals (*n* = 3 mice) and analyzed on different days, normalized to the untreated control. (**B**) LDH assay in LPS-primed macrophages at 4 h post-infection with *C. albicans*. Dot plot shows individual points that represent the mean value of technical replicates from individual experiments using macrophages generated from different animals, and the line represents the mean. Box-and-whisker plot (shown as min-to-max) shows data from a representative experiment (*n* = 3 technical replicates) and analyzed by unpaired *t*-test (*P < 0.05).

Since disrupted phagocytosis and/or phagosome maturation did not explain the killing defect in vancomycin-treated macrophages, we next examined the viability of macrophages following *C. albicans* infection. Using flow cytometry, we observed increased binding of annexin V and uptake of cell viability dyes by vancomycin-treated macrophages compared to untreated macrophages after 1 h of *C. albicans* infection (Fig. 3A). Since macrophage cell death during *C. albicans* infection is linked with inflammasome activation, we next primed the macrophages and examined release of LDH, a marker for pyroptotic cell death, after *C. albicans* infection. These experiments showed that vancomycin-treated macrophages had greater LDH release after *C. albicans* infection (Fig. 3B). Taken together, these data show that vancomycin-treated macrophages are more likely to die following *C. albicans* infection, which may account for their poor fungal killing capacity.

### Vancomycin treatment increases expression of inflammatory and oxidative stress genes during fungal infection

**Fig 4:**
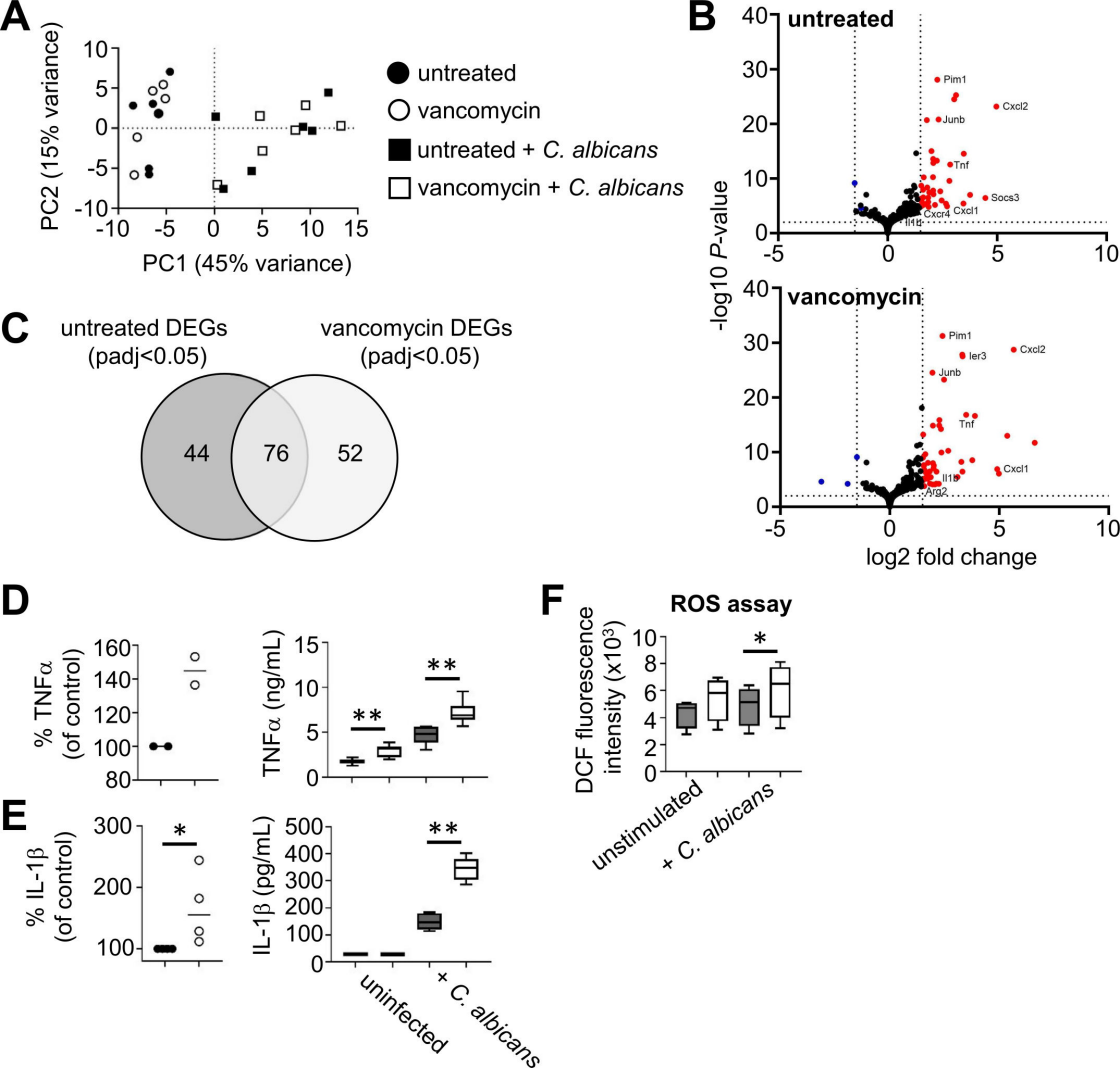
Vancomycin-treated macrophages are more pro-inflammatory. (**A**) PCA plot of untreated and vancomycin-treated macrophages that were either untreated (circles) or infected with live *C. albicans* for 2 h (squares) prior to bulk RNA sequencing analysis. Each point represents an individual experiment using macrophages generated from individual animals (*n* = 6 total). (**B**) Volcano plots showing upregulated and downregulated genes with *C. albicans* infection for untreated macrophages (top) and vancomycin-treated macrophages (bottom). Significant upregulated genes are colored in red, with selected genes labeled. (**C**) Venn diagram comparing the number of unique (44 untreated, 52 vancomycin) and shared (76 genes) DEGs between baseline and infection for untreated macrophages (left) and vancomycin-treated macrophages (right). (**D**) TNFα and (**E**) IL-1β production by untreated and vancomycin-treated macrophages following infection with live *C. albicans*. The dot plot graphs show mean values for individual experiments (where each dot represents macrophages generated from individual mice; the line represents mean), expressed as percentage relative to infected untreated macrophages. The box-and-whisker graphs (shown as min-to-max) on the right show technical replicates from an example experiment, showing baseline and post-infection cytokine production. Data analyzed by Mann-Whitney *U*-test (pooled data, normalized) or two-way ANOVA (example data). (**F**) ROS production assay using H2DCFDA, at baseline and at 3 h post-infection with heat-killed *C. albicans*. Data are pooled from three independent experiments and analyzed by two-way ANOVA. *P < 0.05, **P < 0.01.

Next, we sought to broadly analyze the impact of vancomycin treatment on macrophage responses to *C. albicans* infection using bulk RNA sequencing to better understand mechanisms that may lead to increased death and poor fungal control after vancomycin treatment. We compared untreated and vancomycin-treated macrophages that were either unchallenged or infected with live *C. albicans*. We chose to focus on an early time point post-infection (2 h) to circumvent significant cell damage and death caused by *C. albicans* that occurs after this time point (17). Comparison of uninfected control with uninfected vancomycin-treated macrophages revealed that vancomycin treatment itself did not cause significant transcriptional changes (Fig. 4A and Table S1), in line with our earlier observations that vancomycin treatment did not impair the differentiation or general health of macrophages (Fig. 1). Upon *C. albicans* infection, we found most substantial transcriptional changes were shared between untreated and vancomycin-treated macrophages (Fig. 4B, Table S2), including significant upregulation of immediate-early response genes (*Ier3, Junb*), inflammatory immune signaling (*Nfkbia, Pim1, Irf1*), and cytokines and chemokines (*Cxcl2, Tnf*). We next compared the differentially expressed genes (DEGs) between baseline and infection, comparing untreated and vancomycin-treated macrophages. Interestingly, we found that although 76 genes were shared between untreated and vancomycin-treated macrophages, there were 44 genes that were uniquely differentially regulated in untreated control macrophages during infection, and 52 genes that were uniquely differentially regulated in vancomycin-treated macrophages during infection (Fig. 4C and Table S3). Genes significantly upregulated by control macrophages (but not vancomycin-treated macrophages) included pro-inflammatory surface receptors and transcription factors (*Cd28, Cxcr4, Rora*), glycolytic enzymes (*Eno1, Pfkfb3*), and enzymes involved in biosynthesis of lysosomes and regulation of lipid metabolism (*Tfeb, Olr1, Tmem189*). In contrast, vancomycin-treated macrophages had significant upregulation of genes involved with ubiquitin and oxidative stress responses (*Ubc, Mdm2, Sod2*) and anti-inflammatory genes (*Arg2, Mir155hg, Macir, Nr4a1*). Moreover, we found that vancomycin-treated macrophages had increased expression of *Il1b* and *Nlrp3*, both components of the canonical inflammasome pathway. These results suggested that although vancomycin-treated macrophages have a similar transcriptional response to control macrophages, they have increased expression of inflammatory pathways associated with the inflammasome and concomitant expression of genes involved with dampening inflammation and oxidative stress following infection with *C. albicans*.

To validate our sequencing data, we first analyzed production of inflammatory cytokines TNFα and IL-1β to assess the inflammatory status of macrophages and activation of the inflammasome pathway, respectively. In line with our sequencing data, we found significantly increased production of TNFα by vancomycin-treated macrophages at baseline and following *C. albicans* infection (Fig. 4D). We found a similar increase in IL-1β production by primed vancomycin-treated macrophages (Fig. 4E), in line with our observations of increased LDH release by these cells after infection (Fig. 3B). As we had noted an increase in transcripts encoding oxidative stress response genes in vancomycin-treated macrophages, we also examined production of ROS in our macrophages. We found a significant increase in ROS production in vancomycin-treated macrophages following infection with *C. albicans* (Fig. 4F). Taken together, these data show that vancomycin-treated macrophages have increased production of ROS and inflammatory cytokines following *C. albicans* infection, which may lead to an accelerated death rate under stress.

### Vancomycin causes hyper-fragmentation of mitochondria

**Fig 5:**
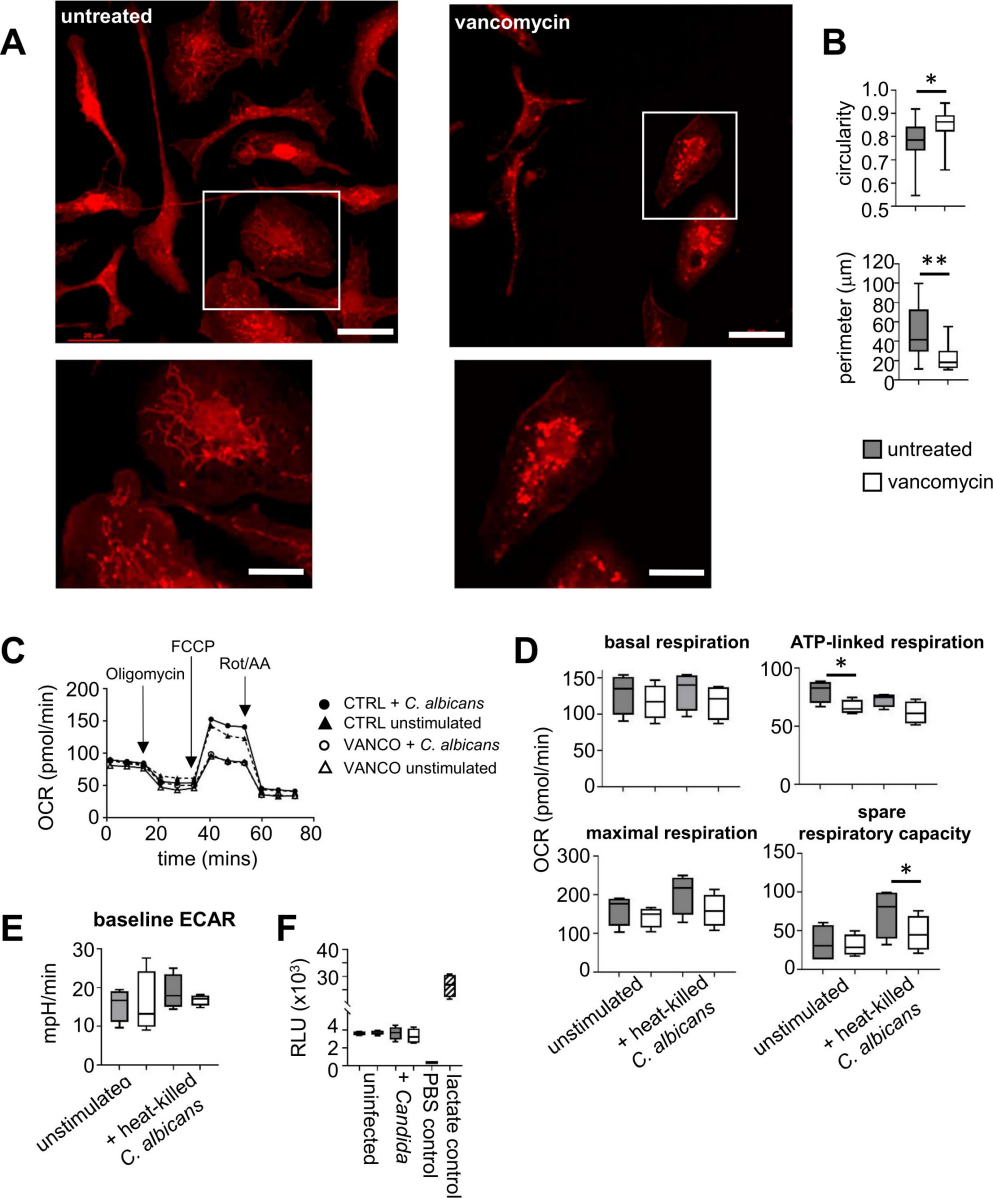
Mitochondria are hyper-fragmented following vancomycin treatment. (**A**) Untreated and vancomycin-treated macrophages were stained with MitoTracker Red to visualize mitochondrial morphology and imaged using confocal microscopy. (**B**) Images were quantified to determine the circularity of mitochondria and perimeter of mitochondria. Plots show pooled data analyzing 200–500 macrophages from three individual experiments. *P < 0.05, **P < 0.01. (**C**) Example Seahorse trace from an individual experiment comparing unstimulated macrophages or macrophages stimulated with heat-killed *C. albicans*. (**D**) Quantification of Seahorse data pooled from four individual experiments and analyzed by paired *t*-tests, *P < 0.05. (**E**) Baseline ECAR determined by Seahorse assay. Data pooled from four individual experiments. (**F**) Lactate release assay comparing macrophages at baseline or at 4 h post-infection with live *C. albicans*. Data are from a single representative experiment (four technical replicates) that was repeated two times. All box-and-whisker plots shown as min-to-max.

Macrophage activation and antimicrobial responses are intimately linked with mitochondrial function (18), and some antibiotics have been shown to exert direct effects on eukaryotic cells via the mitochondria (8). Mitochondrial dysfunction has also been linked with inappropriate inflammation and oxidative stress in epithelial cells (8). Since we observed upregulated expression of genes involved with the regulation of inflammation (including mitochondrial-anchored enzymes *Arg2* and *Mir155hg*) and enhanced ROS production in vancomycin-treated macrophages following *C. albicans* infection (Fig. 4), we next analyzed how mitochondria are affected by vancomycin treatment in macrophages. First, we used confocal microscopy to assess the morphology of mitochondria. After vancomycin pre-treatment, we observed a significant fragmentation of mitochondria and loss of tubular morphology (Fig. 5A). Mitochondria from vancomycin-treated macrophages were smaller and more rounded compared to those in control macrophages (Fig. 5B). These changes were not observed in macrophages early during differentiation and only occurred after 5 days of differentiation in the presence of vancomycin (Fig. S2).

To determine the metabolic consequences of these morphological changes, we analyzed mitochondrial respiration using extracellular flux analysis (“Seahorse assay”), comparing untreated and vancomycin-treated macrophages at baseline or when stimulated with heat-killed *C. albicans* (Fig. 5C). Heat-killed yeast was necessary here, since we wanted to isolate our metabolic analysis to the macrophages, and Seahorse analyzers are not able to distinguish between metabolic signatures of two live cell types (i.e., macrophages and yeast) in the same wells. Our experiments revealed a significant reduction in ATP-linked respiration by vancomycin-treated macrophages, but otherwise baseline respiration appeared similar to that of untreated macrophages (Fig. 5D). Upon stimulation with *C. albicans*, we found a significant reduction in the spare respiratory capacity of vancomycin-treated macrophages (Fig. 5D). To further examine mitochondrial function, we measured abundance of metabolites of the citric acid cycle before and after stimulation with heat-killed *C. albicans*. Those experiments showed similar abundance of metabolites between untreated and vancomycin-treated macrophages (Fig. S3), indicating that activity of the citric acid cycle was not impaired by vancomycin treatment, in agreement with unaltered basal respiration. We also found no difference in baseline extracellular acidification rate (ECAR) and lactate secretion in vancomycin-treated macrophages (Fig. 5E and F), indicating that glycolytic function was unaffected by vancomycin. Taken together, these data show that vancomycin significantly alters mitochondrial morphology in macrophages and their oxidative capacity when challenged with *C. albicans*.

### Vancomycin is observed close to the mitochondria outer membranes

**Fig 6:**
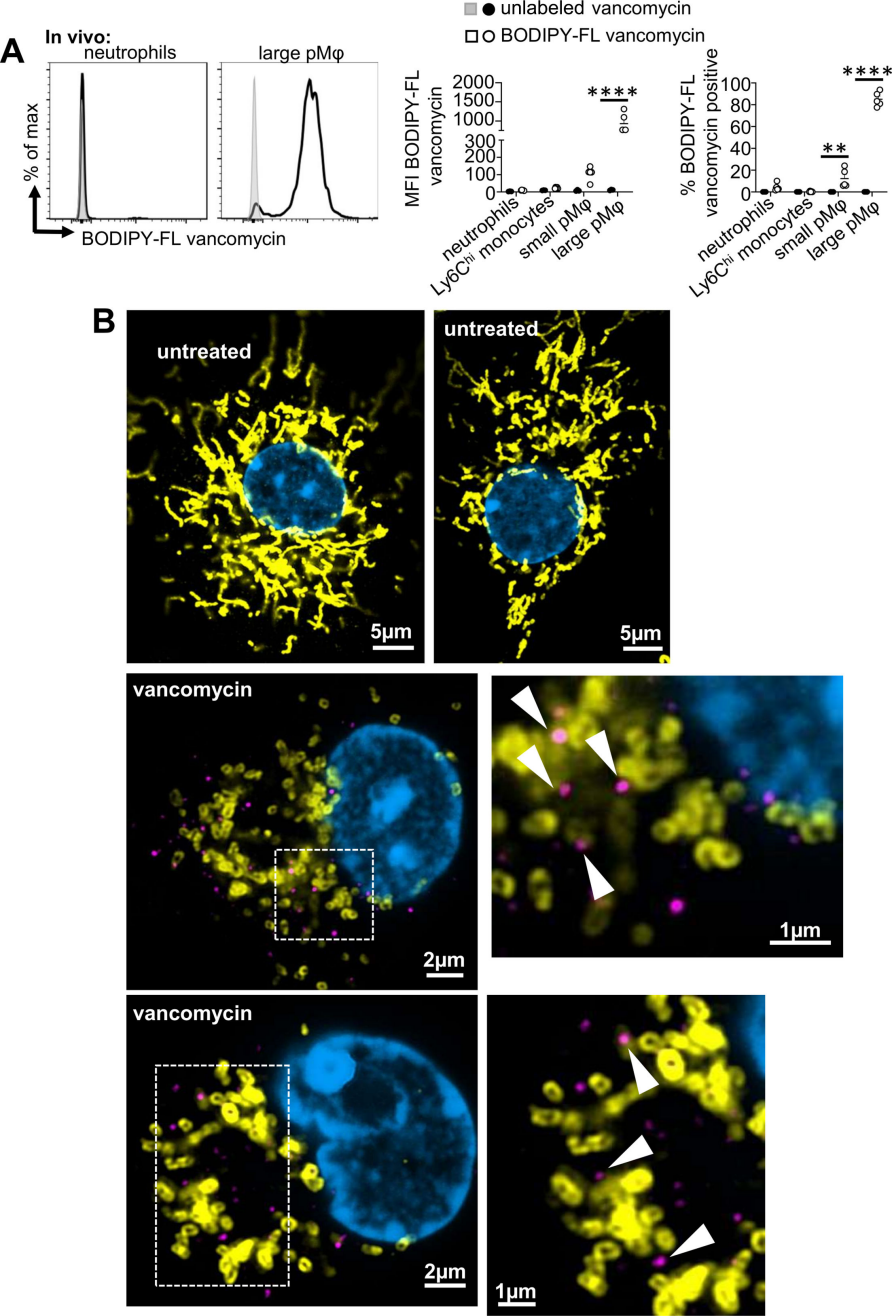
Vancomycin is found in close proximity to the mitochondrial outer membrane. (**A**) Mice were injected intraperitoneally with BODIPY-FL vancomycin and myeloid cells in the peritoneal cavity analyzed by flow cytometry 4 h later. Example histograms show labeling of neutrophils (Ly6G^hi^CD11b^+^) and large peritoneal macrophages (F480^hi^CD11b^hi^), compared with mice injected with unlabeled vancomycin. MFI and frequency of vancomycin labeling are shown for the different populations of myeloid cells in the peritoneal cavity. Each dot represents an individual mouse analyzed (*n* = 5 mice total). Data pooled from two independent experiments, the line represents the mean. Data analyzed by two-way ANOVA. **P < 0.01, ****P < 0.0001. (**B**) Example confocal microscopy images of macrophages stained with mitochondrial marker TOM20 (yellow) and DAPI (blue), either untreated (top row) or treated with BODIPY-FL vancomycin (20 µg/mL for 4–5 h; shown in purple). Areas denoted within dotted lines are shown to the right of the main images, enlarged for clarity. Vancomycin molecules in close proximity to the mitochondria are highlighted with white arrows. Images are shown for two macrophages per condition, which are representative of 10 (untreated) and 9 (vancomycin) cells imaged and analyzed.

We next sought to understand how vancomycin might be exerting its effects on mitochondria. First, we injected mice intraperitoneally with a fluorescently labeled vancomycin and assessed uptake of the antibiotic by macrophages. Within 4 h, we found that macrophages in the peritoneal cavity became positive for the fluorescently labeled vancomycin, particularly the large peritoneal macrophage (F480^hi^CD11b^hi^) population (Fig. 6A). To determine the localization of vancomycin within macrophages, we exposed macrophages to fluorescently labeled vancomycin and used confocal microscopy to observe the cellular localization of vancomycin. We found that fluorescent vancomycin molecules were in close proximity to mitochondria (Fig. 6B). In untreated macrophages, mitochondria were long and extensively branched (Fig. 6B, top row). In macrophages exposed to the fluorescent vancomycin, mitochondria were shorter and more fragmented (Fig. 6B), as we observed previously with the non-fluorescent vancomycin (Fig. 5A and B). Fluorescent vancomycin appeared to be on the outer mitochondrial membrane and not within the mitochondrial lumen (Fig. 6B, white arrows on enlarged insets). Taken together, these data show that vancomycin can penetrate macrophages and localizes near the mitochondrial outer membrane.

### ROS drives mitochondrial depolarization, which is required for fungal killing

**Fig 7:**
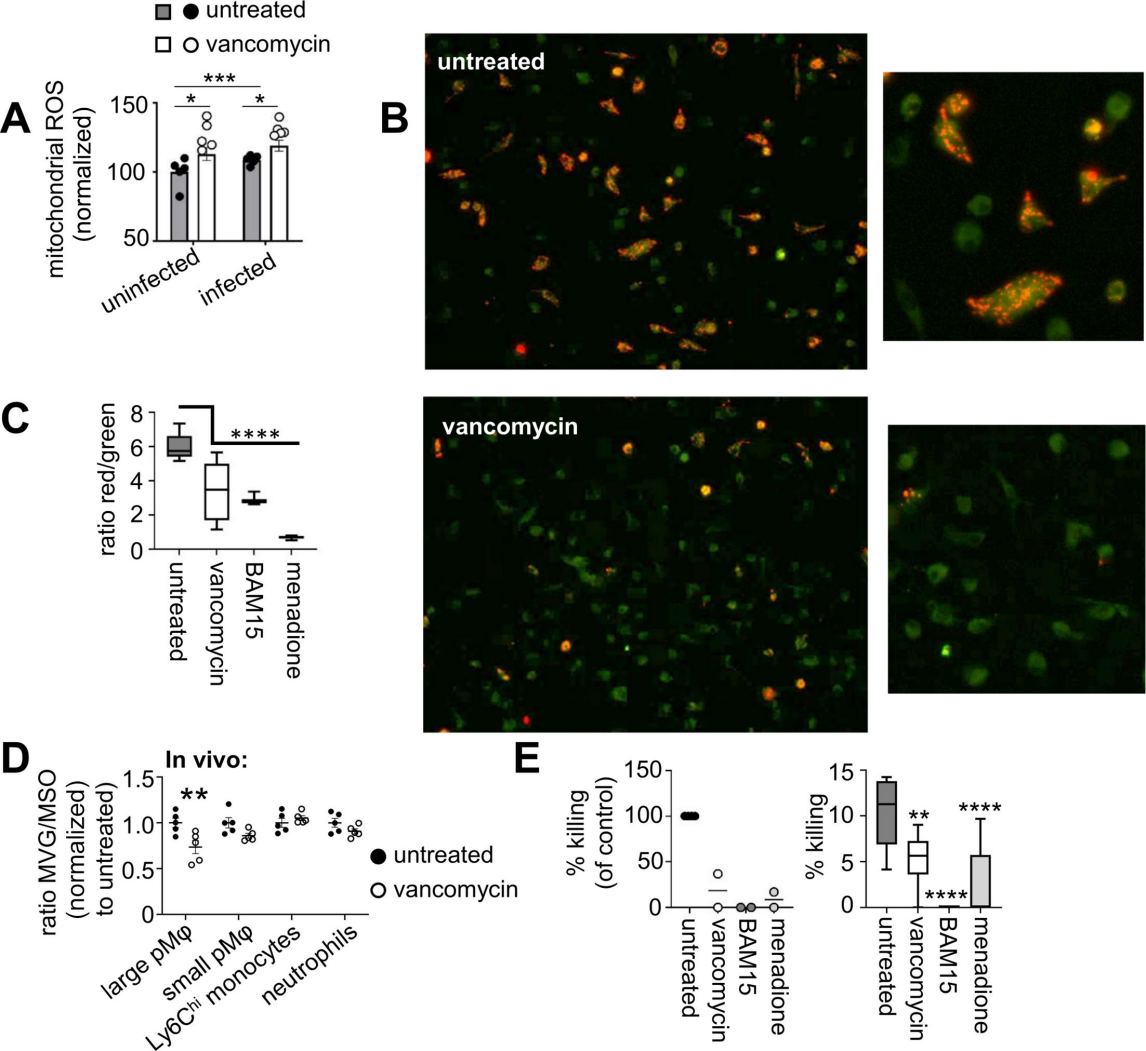
ROS drives mitochondrial polarization, which is required for fungal killing. (**A**) Mitochondrial ROS staining by MitoSOX in untreated and vancomycin-treated macrophages at baseline or at 2 h post-infection with live *C. albicans*. Bar graphs show mean ± SEM for four independent experiments (using macrophages generated from different animals); overlaid dot plots show technical replicate values for one of these experiments. Data analyzed by two-way ANOVA (on example experimental data). *P < 0.05, ***P < 0.005. (**B**) Example JC-1 labeling of untreated and vancomycin-treated macrophages. (**C**) Ratio of red and green JC-1 labeling in untreated and vancomycin-treated macrophages, compared to untreated macrophages treated with BAM15 for 2 h as a positive control and untreated macrophages treated with 5 mM menadione for 2 h. Data pooled from two independent experiments in which at least 200 macrophages were analyzed. Data analyzed by one-way ANOVA, comparing to untreated macrophages. Box-and-whisker plots shown as min-to-max. ****P < 0.0001. (**D**) Ratio of MitoView Green (MVG) and MitoSpy Orange (MSO) staining of indicated myeloid cells in the peritoneal cavity of mice either untreated or treated intraperitoneally with vancomycin. Data normalized to the untreated animals. Each point represents an individual animal analyzed (*n* = 5 total), pooled from two independent experiments. Data shown as mean ± SEM; analyzed by two-way ANOVA. **P < 0.01. (**E**) Fungal killing assay using untreated, vancomycin-treated, BAM15-treated, and menadione-treated macrophages. Dot plot shows a point representing the mean of technical replicates of individual experiments using macrophages generated from different animals, and the line represents the mean. Box-and-whisker (shown as min-to-max) plot shows a representative experiment with *n* = 10 technical replicates and analyzed using one-way ANOVA, comparing to untreated macrophages. **P < 0.01, ****P < 0.0001.

Hyper-fragmented mitochondria have previously been associated with oxidative stress and reduced membrane potential and increased ROS generation, linked to prolonged electron dwell time at complexes I and III (19, 20). In line with that, we found increased ROS production by these cells and increased expression of oxidative stress genes (Fig. 4). To determine if these phenotypes are linked, we first measured the production of mitochondrial-specific ROS in untreated and vancomycin-treated macrophages using mitoSOX staining. These experiments demonstrated that vancomycin treatment induced significantly more mitochondrial ROS both at baseline and during infection (Fig. 7A). Oxidative stress, caused by over-production of ROS, can occur at both extremes of mitochondrial membrane potential as the cell attempts to balance antioxidant and oxidative redox couples (21). We therefore examined the impact of vancomycin treatment on mitochondria membrane potential (ψm) using JC-1 dye, which exhibits a red fluorescence when accumulated within polarized mitochondria and a green fluorescence within non-polarized mitochondria (22). Using this approach, we found that vancomycin significantly dissipated ψm (lower red/green ratio) when compared to control macrophages (Fig. 7B and C). Indeed, vancomycin-treated macrophages had similar ψm to untreated macrophages exposed to the mitochondrial uncoupler BAM15, and to macrophages treated with the ROS inducer menadione, which had a significantly decreased ψm (Fig. 7C). To examine whether similar mitochondrial depolarization occurred *in vivo*, we intraperitoneally injected mice with vancomycin and used mitochondrial mass and ψm indicator dyes to assess mitochondrial health in peritoneal myeloid cells using flow cytometry. We found that large peritoneal macrophages had significantly reduced ψm in vancomycin-exposed animals compared to untreated mice, based on a reduced ratio between the ψm-dependent dye and mitochondrial mass dye (Fig. 7D). Indeed, our earlier data indicated that this macrophage population was the predominant one to become labeled with vancomycin (Fig. 6A) and may therefore be more sensitive to effects of the antibiotic.

We next directly probed the relationship between mitochondrial depolarization and fungal killing by macrophages. For that, we pre-treated macrophages with either BAM15 or menadione and then challenged them with live *C. albicans*. These experiments showed that macrophages were impaired in fungal killing when treated with either of these drugs, similar to vancomycin-treated macrophages (Fig. 7E). However, vancomycin-treated macrophages treated with mitochondrial ROS inhibitors did not exhibit improved fungal killing (Fig. S4), indicating that mitochondrial ROS by itself is not sufficient to drive the fungal killing defect in these cells. Taken together, these data show that impaired fungal killing by vancomycin-treated macrophages is linked to the depolarization of their mitochondria.

## DISCUSSION

The immune system is sensitive to antibiotic treatment. Multiple studies have now shown that prior antibiotic exposure affects the ability of the mammalian immune system to fight infections (7, 23, 24). Here, we explored the direct effects of vancomycin on antifungal immune responses, focusing on macrophages. We found that vancomycin impacted the metabolic capacity and inflammatory function of macrophages. Vancomycin-treated macrophages had fragmented, depolarized mitochondria and impaired respiratory capacity when exposed to *C. albicans*. These changes to mitochondrial function and oxidative stress are associated with a decreased capacity of macrophages to kill *C. albicans* fungi.

Vancomycin has been previously shown to disrupt autophagy pathways, resulting in a build-up of LC3 (12). LAP is a specialized phagocytosis in which components of the autophagy pathway are re-purposed for microbial uptake, including *C. albicans*. We therefore explored general phagocytic ability and abundance of LC3 in vancomycin-treated macrophages to determine whether LAP of *C. albicans* was disrupted by vancomycin treatment. While we found a general phagocytosis defect in vancomycin-treated macrophages when challenged with fluorescent particles made from *E. coli* bacteria, we did not find any impairment of uptake of live *C. albicans* nor any difference in LC3 abundance with antibiotic treatment. Several antibiotics have been reported to disrupt phagocytosis processes, including ciprofloxacin and ampicillin (9, 10), but whether this occurs via LC3 is unknown, or whether the methods used to harvest macrophages affect interpretation of these results. Previous work found that LC3 is readily recruited to *C. albicans*-containing phagosomes (14), and LC3-deficient macrophages are impaired in their *C. albicans* killing capacity and were more inflammatory, indicated by greater production of TNFα and IL-1β when stimulated with *C. albicans* yeast (25). Recruitment of LC3 to *C. albicans*-containing phagosomes depended on recognition of β-glucan via dectin-1 and ROS generation but did not require TLR signaling (25). However, *C. albicans* has also been shown to inhibit LC3 turnover within macrophages and may interfere with deposition of this protein on phagosomes (26). Indeed, LC3 recruitment to phagosomes is increased in macrophages that have phagocytosed heat-killed yeast compared to live yeast (25). In line with these findings, we observed an increase in LC3 abundance in macrophages infected with *C. albicans*, but this was unaffected by vancomycin treatment. Therefore, while vancomycin may impair phagocytosis generally, this may be less relevant for the fungal pathogen *C. albicans*, which is able to alter the dynamics of LC3 deposition on phagosomes. Whether vancomycin impairs other specific phagocytosis pathways that are independent of LC3, and if this has an impact on uptake of other types of pathogens (e.g., bacteria), will be important to determine the relevance of phagocytic defects caused by vancomycin in susceptibility to other types of infection.

In contrast to phagocytosis, we found a consistent and profound impact on the mitochondria of vancomycin-treated macrophages. Vancomycin was observed in close proximity to mitochondria, which had a significantly altered morphology associated with depolarization and increased ROS production. Mitochondrial respiration and remodeling help determine myeloid cell function. Macrophages responding to LPS undergo a switch from oxidative phosphorylation to glycolysis to generate energy, resulting in a rewiring of the electron transport chain and significant changes to mitochondrial function (18). Indeed, the ability to dynamically alter mitochondrial respiration in response to stimuli and cytokines is required to support myeloid functions such as antigen presentation (27), tissue repair (28), and clearance of bacterial infection (29). Using fluorescently labeled vancomycin as a probe, we found that vancomycin appeared to closely align with the mitochondrial outer membrane. Previous work that examined vancomycin binding to liposomes showed that the lipid PG had the greatest affinity for vancomycin binding, but that binding capacity was highly dependent on the head groups of the lipids and the curvature of membrane (30). PG is transported to the inner mitochondrial membrane and acts as a precursor for cardiolipin biosynthesis, a lipid found exclusively within mitochondria. PG is a relatively rare lipid species in mammalian membranes and is more abundant in bacterial membranes (31). Vancomycin may therefore localize to mitochondria in mammalian cells via the increased abundance of PG used for cardiolipin synthesis. The binding of vancomycin to other lipid types in different cell types will be required to determine how this antibiotic penetrates immune cells and the extent to which this affects function of different organelles. This may be particularly pertinent in cell types that produce high levels of ROS and undergo cell death readily during fungal infection, such as neutrophils. Neutrophils also demonstrate sensitivity to some antibiotics (23, 32) and are a critical component in effective immunity against *C. albicans* (11).

In addition to mitochondrial defects, we also observed that vancomycin-treated macrophages were more inflammatory than their untreated counterparts, producing more TNFα and IL-1β when exposed to *C. albicans*, resulting in divergent inflammatory phenotypes. We also noted an increase in expression of oxidative stress genes with vancomycin treatment, which correlated with enhanced ROS production by these cells. This aligns with previous observations made with renal tubular cells, which were more apoptotic due to increased mitochondrial ROS production following exposure to vancomycin (33). Indeed, we also observed increased cell death in vancomycin-treated macrophages infected with *C. albicans*. The specific pathways driving antibiotic-mediated inflammatory cell death remain to be determined, yet this remains an important future avenue for investigation to better understand the influence of antibiotics on immune cell health and viability.

Interestingly, our RNA sequencing analysis revealed an upregulation of multiple anti-inflammatory genes in vancomycin-treated macrophages upon infection that we did not observe in untreated macrophages. These included the enzyme arginase-2 and nuclear receptor Nr4a1. Arginase-2 is an anti-inflammatory enzyme anchored to the mitochondria and responsible for downregulating IL-1β and HIF1α signaling in response to IL-10 stimulation (34). Arginase-2 is further regulated by microRNA-155 (34), which we also found was significantly increased in vancomycin-treated macrophages upon *C. albicans* infection. Despite being considered an anti-inflammatory enzyme, arginase-2 has been shown to act in a pro-inflammatory capacity in some contexts primarily through mitochondrial ROS (35), aligning with some of the observations we have made in this study. Furthermore, we found that vancomycin treatment upregulated expression of Nr4a1 (also known as Nur77), which plays an integral role in limiting macrophage inflammatory responses via its role in mitochondrial respiration. Nr4a1-deficient macrophages have greater production of IL-1β and IL-6 that was associated with a reduction in oxidative phosphorylation and a break in the citric acid cycle leading to a build-up of succinate (36).

Although we observed significant effects of vancomycin treatment on inflammatory phenotype and mitochondrial function, we did not observe any significant change in glycolysis. Macrophages switching to glycolysis for energy production is known as the Warburg effect and has been observed during several inflammatory conditions (37). Indeed, other work has shown that macrophages switch to glycolysis and upregulate pathways involved in glucose uptake during *C. albicans* infection, with this metabolic switch rapidly increasing from 3 h of infection and peaking at 6–9 h post-infection (17). These studies analyzed infection times that were much longer than in the current study, in which we focused on the earlier time point of 2 h post-infection as we wished to examine the broad effects of vancomycin on macrophage function and initial interactions with *C. albicans* yeast. The upregulation of glucose uptake and dependency on glucose for energy production by *C. albicans*-infected macrophages plays a central role in the death of these cells during infection (17). The fungus was also found to upregulate glucose uptake pathways when exposed to macrophages, leading to a metabolic competition between macrophages and *C. albicans*, resulting in glucose starvation and death of macrophages once *C. albicans* numbers sufficiently outcompeted the macrophages (17). Indeed, glucose starvation in *C. albicans*-infected macrophages was found to activate the NLRP3 inflammasome and IL-1β production and subsequent inflammatory cell death (pyroptosis). Macrophage death following *C. albicans* infection did not require formation of hyphae by *C. albicans* but was highly strain-dependent (38). Indeed, the fungal toxin candidalysin, produced by *C. albicans*, is a potent inflammasome activator (39), but its production and release varies widely between *C. albicans* clinical isolates (40). In our work, we found that vancomycin treatment appeared to accelerate inflammasome activation during *C. albicans* infection, resulting in increased IL-1β production and a higher rate of macrophage death. These effects may be occurring independently of metabolic competition for glucose, since we did not observe changes in ECAR or expression of genes involved in glucose uptake between untreated and vancomycin-treated macrophages, although we cannot rule out effects on other types of metabolic competition that may be occurring between macrophages and *C. albicans*. Future studies should aim to examine how competition for other sources of energy between macrophages and *C. albicans* (e.g., lipids) fuels infection-relevant phenotypic changes, and how these may be affected in the context of antibiotic treatment.

In summary, we have found functional defects in immune cells exposed to vancomycin that impaired the ability to fight pathogenic fungus *C. albicans*. Vancomycin enhanced inflammatory pathways in macrophages and directly impaired mitochondrial function, which was required for optimal fungal killing by macrophages.

## MATERIALS AND METHODS

### Mice

Eight- to 12-week-old C57BL/6JCrl mice (males and females) were housed in individually ventilated cages under specific pathogen-free conditions at the Biomedical Services Unit at the University of Birmingham and had access to standard chow and drinking water *ad libitum*. In some experiments, drinking water was supplemented with 0.5 mg/mL vancomycin hydrochloride, and this water was refreshed twice per week. In some experiments, mice were injected with vancomycin intraperitoneally, given as three separate doses (200 µg per dose) 48 h apart. Mice were analyzed 24 h after the last dose. Mice were housed under a 12 h light/dark cycle at 20°C–24°C and 45%–65% humidity.

### *C. albicans* growth and *in vitro* infections

*C. albicans* strains used in this study were SC5314 and SC5314-dTomato. Yeast was routinely grown in YPD broth (2% peptone [Fisher Scientific], 2% glucose [Fisher Scientific], and 1% yeast extract [Sigma]) at 30°C for 18 h at 200 rpm. For infections, yeast cells were washed twice in sterile PBS, counted using a hemocytometer, and diluted to required concentrations. In general, live yeast were added to cultured macrophages at a ratio of 2:1 (yeast:macrophages) unless otherwise stated. In some experiments, yeast were heat-killed prior to use in experiments. Heat-killing was performed by incubating washed yeast cultures at 70°C for 30 min. Heat-killed yeast were used immediately following cooling on ice.

### Mouse *C. albicans* infection model

Mice were infected intravenously with 1 × 10^5^ yeast cells, prepared as above, via the lateral tail vein. Mice were monitored daily for weight change and development of clinical symptoms (e.g., hunched posture, hypothermia) and were euthanized by cervical dislocation at indicated analysis time points, or when humane endpoints (e.g., 20%–25% weight loss, hypothermia) had been reached, whichever occurred earlier. For analysis of tissue fungal burdens, animals were euthanized and organs weighed, homogenized in PBS, and serially diluted before plating onto YPD agar supplemented with penicillin/streptomycin (Invitrogen). Intestines were first thoroughly washed in PBS prior to homogenization and plating. Bacterial burdens from the spleen were measured as above, except homogenates were plated onto sheep blood agar (Remel). Colonies were counted after incubation at 37**°**C for 24–48 h.

### Differentiation of bone-marrow macrophages

Bone marrow was flushed from the femurs and tibias of mice (8- to 12-week-old C57BL/6JCrl males and females) into sterile PBS supplemented with 2 mM EDTA. Collected bone marrow was filtered through a 70 μm filter and centrifuged (1,500 rpm, 5 min at 4°C) to pellet the cells. The pellet was resuspended in Roswell Park Memorial Institute (RPMI) media with GlutaMAX and HEPES, further supplemented with 20% heat-inactivated fetal bovine serum and 40 ng/mL M-CSF (BioLegend). Half of the cell suspension was further supplemented with 20 μg/mL vancomycin hydrochloride (Fisher BioReagents). The cell suspension (10 mL per flask) was incubated in T75 flasks (Corning) for 3 days at 37°C at 5% CO_2_, before removing the media and replacing it with 10 mL fresh media (supplemented with FBS and M-CSF with/without vancomycin, as above), before continuing the incubation for a further 2 days. On day 5 of the culture, adherent macrophages were collected by harvesting in ice-cold 2 mM EDTA/PBS. Briefly, the media was removed and cell layer flooded with ice-cold 2 mM EDTA/PBS before incubation on ice for 5 min. Cells were gently lifted with a cell scraper (Corning) and collected by centrifugation. Macrophages were counted using a hemocytometer and Trypan blue exclusion. Macrophages were cultured in RPMI supplemented with 10% FBS without antibiotics or with 20 μg/mL vancomycin in subsequent assays.

### Macrophage functional polarization assays

Following differentiation, macrophages were harvested as above and seeded into 24-well plates at 1 × 10^6^ macrophages per well. Macrophages were then cultured as above in complete RPMI (“M0”), or RPMI supplemented with 100 ng/mL LPS and 20 ng/mL recombinant murine IFNγ (BioLegend) for “M1,” or 10 ng/mL recombinant murine IL-4 (BioLegend) for “M2.” Macrophages were harvested and analyzed by flow cytometry after 24 h of stimulation.

### Flow cytometry

Cultured macrophages (harvested from plates or tissue culture flasks) or peritoneal lavage fluid from mice were placed in FACS tubes for staining. Samples were washed in FACS buffer (PBS supplemented with 0.01% sodium azide and 2.5% wt/vol bovine serum albumin), and stained with fixable viability dye (ZombieViolet, BioLegend) as per manufacturers’ instructions. Fc receptors were blocked with anti-CD16/32 (24G2). Fluorescently conjugated antibodies were then added to the samples for 15–60 min on ice, protected from the light. Samples were washed in FACS buffer and then either acquired immediately or fixed in 2% paraformaldehyde prior to acquisition. In some experiments, samples were fixed/permeabilized using the Foxp3 transcription factor staining buffer set (eBioscience) prior to staining for intracellular antigens, overnight at 4°C. In some experiments, samples were washed with annexin-V binding buffer prior to labeling with annexin V-APC (BioLegend) for 20 min at room temperature, before immediate acquisition. Anti-mouse antibodies used in this study were: CD11b-APC-Cy7 (BioLegend, clone: M1/70), F4/80-APC (BioLegend, clone: BM8), MHCII-PE-CY7 (BioLegend, clone: M5/114.15.2), iNOS-APC (Invitrogen, clone: CXNFT), arginase-PE (BioLegend, clone: W21047I), Ly6G-PerCP Cy5.5 (BioLegend, clone 1A8), Ly6C-AF700 (BioLegend, clone: HK1.4). Antibodies were used at 2 µg/mL. All samples were acquired using a 5-laser BD LSR Fortessa equipped with BD FACSDiva software. Data analysis was performed using FlowJo software v.10.9.0 (TreeStar).

### MitoView Green and MitoSpy Orange staining (flow cytometry)

Peritoneal lavages from mice were centrifuged to pellet cells (300 g, 5 min, 4°C), and the cell pellets resuspended in 100 µL of RPMI media supplemented with 10% heat-inactivated FBS. Cells were rested for 20 min at 37°C. Metabolic probes MitoSpy Orange (25 nM, BioLegend) and MitoView Green (50 nM, Biotium) were added to the cells and incubation continued for 30 min at 37°C. Then cells were washed in PBS and then stained for surface markers as described above.

### Phagocytosis assays

For *C. albicans* phagocytosis assay, macrophages were lifted and counted as above prior to seeding in 24-well plates (2 × 10^5^ macrophages per well) and rested overnight. Prepared dTomato-expressing *C. albicans* was then added to the macrophages (at an MOI of 2:1) the following day. In some experiments, *C. albicans* was opsonized by incubating the yeast cells with 10% mouse serum (Thermo Fisher) on ice for 30 min prior to adding to the macrophages. Incubation of the infected macrophages was performed at 37°C and was continued for 30 min or 1 h, before immediately placing plates on ice to stop phagocytosis. Macrophages were then lifted using 2 mM EDTA/PBS as above, and then stained with the following fluorophore-conjugated antibodies on ice for 15–30 min in the dark: anti-mouse CD45-PerCP-Cy5.5 (clone 30-F11), anti-mouse CD11b-APC-Cy7 (clone M1/70), anti-mouse F4/80-APC (clone BM8) (all BioLegend) and anti-Candida albicans-FITC (Invitrogen). Samples were then washed in FACS buffer (PBS supplemented with 0.01% sodium azide and 2.5% wt/vol bovine serum albumin) and acquired using a 5-laser BD LSR Fortessa equipped with BD FACSDiva software. Data analysis was performed using FlowJo software v.10.9.0 (TreeStar). For analysis of general phagocytic function, macrophages were seeded into 96-well flat-bottom plates (1 × 10^5^ macrophages per well) as above. Phagocytosis was assessed using the Vybrant phagocytosis assay kit (Thermo) following the manufacturer’s instructions, reading the plate fluorescence at 480/520 nm excitation/emission using a FlexStation 3 plate reader equipped with SoftMax Pro 7 software.

### Fungal killing assay by Alamar Blue viability

Macrophages (5 × 10^4^ per well) were seeded into 96-well plates as above. In some experiments, macrophages were pre-treated with 5 mM menadione, BAM15, mitoQ, or mitoTEMPO for 2 h. Media was removed the following day from all wells and replaced with 50 μL sterile PBS supplemented with 10% mouse serum. Prepared *C. albicans* was then added to each well at an MOI of 2:1, and the cells were incubated at 37°C for 2 h. After the 2-h infection period, the plates were centrifuged (2,000 rpm for 4 min), and supernatants removed. One hundred microliters of 0.02% Triton X-100 in distilled water was added to each well, and the plate was incubated at room temperature for ~5 min. Plates were centrifuged again, the supernatant removed, and each well washed twice with 100 μL PBS. After washing, 100 μL of 1× Alamar Blue metabolic indicator dye (Thermo) was added to each well and the plate incubated at 37°C for 18 h. Plate fluorescence was read at 560/590 nm excitation/emission using a FlexStation 3 plate reader equipped with SoftMax Pro 7 software. The number of yeast per well was calculated by comparing to a standard curve of yeast-only wells, and percentage killing was determined based on the difference between starting number and final number after co-incubation with macrophages.

### Lysotracker red staining and live cell imaging

A total of 2 × 10^5^ macrophages were seeded per well in 24-well plates as above. Medium was replaced the following day with fresh medium containing 50 ng/mL Lysotracker Red DND-99 (Thermo Fisher). Cells were infected with live or heat-killed *Candida* at an MOI of 6:1, and fluorescence was read every 5 min for 6 h on a Zeiss Axio Observer microscope at 40× magnification, fitted with a temperature-controlled chamber set at 37°C with 5% CO_2_.

### RNA sequencing

Macrophages were seeded overnight in 24-well plates at 1 × 10^6^ cells/well and infected with *C. albicans* for 2 h (MOI 2:1) or mock infected with PBS. Media was then removed from wells and 1 mL of Trizol (Life Technologies) added to each well. Lysed cells were lifted from the wells, transferred to RNase-free Eppendorfs, and stored at −80°C. Samples were thawed at room temperature, and 200 μL of chloroform was added to each tube, mixed by inverting several times, and allowed to settle for 5 min. Samples were centrifuged at 12,000 *g* for 15 min at 4°C. The top layer containing RNA was transferred to RNase-free Eppendorf tubes and an equal volume of 70% ethanol was added to each sample. Samples were then added to Qiagen columns (RNeasy kit, Qiagen) to be washed and perform a DNase digest step (Qiagen). RNA was eluted in RNase-free water, and then QC-tested using the Qubit Fluorometer TapeStation. RNA libraries were then prepared using the Lexogen QuantSeq 3′ mRNA-Seq kit according to the manufacturer’s instructions. Illumina sequencing was performed on the NextSeq 500 sequencing platform using the v.2.5 150-cycle mid-output flow cell, which produced 75 bp single reads. Reads were mapped to the *Mus musculus* reference genome. Read counts were quantified using HTSeq-count. Differentially expressed genes between biological conditions were detected using the DESeq2 analysis pipeline in R (*P*_adj_ < 0.05). Sequencing data have been deposited in the NCBI GEO repository under accession number GSE269051.

### ELISAs

Macrophages were seeded overnight in 96-well tissue culture plates at 1 × 10^5^ cells/well prior to infection with *C. albicans* at an MOI of 6:1. In some experiments, macrophages were first primed with 50 ng/mL LPS (Sigma) for 2 h prior to infection (IL-1β assay). Infected macrophages were incubated at 37°C for 4 (IL-1β assay) or 6 h (TNFα assay), and supernatants were collected and frozen at −20°C. Detection of IL-1β and TNFα was performed using DuoSet ELISA kits (R&D Systems) according to the manufacturer’s instructions. Plate absorbance at 450 nm was read using a SpectraMax ABS Plus plate reader, equipped with SoftMax Pro 7 software.

### LDH assay

Supernatants from macrophage cultures (see figure legends for specific conditions) were collected and stored at −20°C. LDH was quantified in the supernatants using the CyQUANT LDH Cytotoxicity Assay Kit (Invitrogen) following the manufacturer’s instructions. Plate absorbance was read at 490 nm and 680 nm within 2 h. LDH activity was calculated by subtracting the 680 nm absorbance value (background signal) from the 490 nm absorbance value.

### Lactate measurements

Lactate was measured in macrophage culture supernatants using the Lactate-Glo Assay (Promega) following manufacturer’s instructions. Luminescence was measured using a FlexStation 3 plate reader equipped with SoftMax Pro 7 software.

### ROS induction and measurements

Intracellular reactive oxygen species were assessed using the H_2_DCFDA probe (Thermo Fisher). Macrophages were seeded overnight in 96-well white opaque plates (Costar) at a concentration of 1 × 10^5^ cells/well. Cells were gently washed with pre-warmed PBS, and H_2_DCFDA at a concentration of 40 μM was added for 30 min at 37°C. BMDMs were then infected with heat-killed *C. albicans* at the indicated multiplicity of infection in PBS supplemented with 10% FBS and incubated at 37°C with 5% CO_2_ for the indicated times. Fluorescence intensity of DCF was measured using a fluorescence spectrophotometer (FlexStation 3 equipped with SoftMax Pro 7 software) at an excitation and emission wavelengths of 485 nm and 525 nm, respectively.

### Confocal microscopy

Macrophages were seeded overnight onto glass coverslips (12 mm) placed within 24-well plates (Corning) for fluorescent imaging at 2 × 10^5^ cells per well. Coverslips were mounted onto glass slides prior to imaging. For JC-1 staining, media was removed from each well and replaced with sterile PBS to wash the macrophages. The PBS was removed and replaced with fresh RPMI media containing JC-1 dye (Thermo) diluted to 1:10,000. Macrophages were incubated with the dye for 30 min at 37°C, then washed again in PBS. Stained macrophages were imaged using an inverted epifluorescence microscope (Olympus IX71) coupled to Retiga R6 CCD digital camera (QImaging, Teledyne), and the red/green ratio of JC-1 labeling quantified using ImageJ software (v.1.54, Fiji). For MitoTracker Red staining, macrophages were seeded onto coverslips as above and stained in pre-warmed RPMI media containing 50 ng/mL MitoTracker Deep Red dye (Thermo) for 30 min at 37°C. Macrophages were then washed and fixed in 4% paraformaldehyde for 30 min at room temperature, protected from light. Macrophages were then stained with DAPI (2 μg/mL) for 30 min at room temperature prior to mounting onto glass slides with ProLong Gold anti-fade mountant (Thermo). Stained macrophages were imaged using the Airyscan function of a Zeiss LSM 880 confocal microscope (Zeiss) using a ×63 water immersion objective.

### Fluorescent vancomycin labeling (*in vitro*)

For vancomycin localization studies, macrophages were seeded and washed as above and then incubated in fresh RPMI media containing 50 µM BODIPY-FL vancomycin (Thermo) at 37°C for 4–5 h. Macrophages were washed and fixed in 4% paraformaldehyde for 10 min at room temperature, followed by permeabilization in 1.5% PBS-Tween for 3 h. Cells were washed three times in PBS and incubated overnight at 4°C with rabbit anti-TOMM20 antibody (HPA011562, Sigma-Aldrich). After washing, cells were incubated with Alexa Fluor 647-conjugated goat anti-rabbit secondary antibody (A21244, Invitrogen) for 1 h at room temperature. Cells were then washed five times in PBS (5 min each) and mounted in Vectashield Plus with DAPI (H-2000-10, Vector Laboratories). Labeled cells were imaged using the Airyscan function of a Zeiss LSM 900 confocal microscope (Zeiss) using a ×63 oil immersion objective.

### Fluorescent vancomycin labeling (*in vivo*)

C57BL/6 females (8–10 weeks old) were injected intraperitoneally with 250 µg BODIPY-FL vancomycin (Thermo) or unlabelled vancomycin as a control. Mice were euthanized after 4 h, and a peritoneal lavage was performed using sterile PBS. Cells within the lavage were stained and analyzed by flow cytometry, as above.

### SDS-PAGE and Western blotting

Macrophages were seeded overnight in 24-well plates as above. Cells were then infected with *C. albicans* at an MOI of 2:1 (yeast : macrophages) and incubated for 4 h. Macrophages were lysed in RIPA buffer supplemented with protease and phosphatase inhibitors (Sigma) on ice for 10 min. Protein concentration of the lysates was measured using the microplate procedure of the Pierce BCA protein assay kit (Thermo) as per the manufacturer’s instructions. Protein lysates were first boiled in Laemmli loading buffer (Sigma) for 5 min at 95°C. Equal amounts of protein were loaded onto 10% acrylamide gels and run at 90 V for 90–120 min. Separated proteins were then transferred from gels to 0.45 µm PVDF membranes (Thermo) using iBlot 2 gel transfer device (Life Technologies). Membranes were incubated in blocking buffer (6% non-fat milk in PBS-Tween-20 or 5% BSA in PBS-Tween-20) for 1 h at room temperature. Membranes were briefly washed in wash buffer (PBS supplemented with 10 mM Tris, 100 mM sodium chloride, and 0.1% Tween-20) and then probed with anti-β-actin or anti-LC3 antibody (Cell Signaling Technologies), diluted 1:1,000 in blocking buffer, overnight at 4°C on a shaker platform. Membranes were washed with washing buffer using three 5 min cycles. Membranes were then probed with mouse anti-HRP (clone 7076s) or rabbit anti-HRP (clone 7074s) secondary antibody (Cell Signaling Technologies) for 2 h at room temperature. This was followed by extensive washing in wash buffer (5 cycles of at least 5 min) and a final wash step in distilled water. Clarity Western ECL Substrate (Bio-Rad) was added to the membranes for 1 min in the dark, prior to imaging with a Gel Doc imaging system (Bio-Rad).

### Extracellular flux (“Seahorse”) assay

Control and vancomycin-treated macrophages were seeded overnight at 37°C at 2 × 10^5^ cells per well (150 μL final volume of antibiotic-free or vancomycin media, as above) in a 96-well Seahorse tissue culture plate (Agilent). The cartridge/utility plate (Agilent) was hydrated by immersing probes in 200 μL of distilled water and incubated overnight in a non-CO_2_ 37°C incubator with adequate humidity. XF Calibrant solution (Agilent) was also pre-warmed in a non-CO_2_ incubator overnight. Heat-killed *C. albicans* was added to each well the following day, at an MOI of 10:1 (yeast:macrophage) and incubation at 37°C continued for a further 4 h. Seahorse media was prepared using XF DMEM base media supplemented with 1 mM sodium pyruvate, 10 mM glucose, and 2 mM L-glutamine (all Agilent). Samples were analyzed on a Seahorse X-96 Flux analyzer equipped with Wave software. Prior to analysis, the analyzer was initialized using the pre-prepared cartridge plate after removing the water and replacing it with pre-warmed calibrant and loading the utility plate with oligomycin, FCCP, and rotenone/antimycin A (all Agilent; Seahorse mitochondrial stress test kit) in the relevant injection ports. The plate containing cells/fungi had media removed and replaced with 180 µL Seahorse media prior to adding to the analyzer. Drugs were injected as per the Stress Test kit program at final concentrations of 1.5 μM oligomycin, 2 μM FCCP, and 0.5 μM rotenone/antimycin A.

### Metabolite screen by LC-MS

Macrophages were seeded into 24-well plates overnight (as above), and then challenged with heat-killed *C. albicans* for 2 h (10:1 fungi to macrophage). Macrophage monolayers were washed with 0.9% sodium chloride solution on ice twice prior to quenching and lifting with 0.5 mL 70% acetonitrile. Macrophages were snap-frozen on dry ice and thawed on wet ice three times prior to spinning down; supernatants were collected (approx. 0.5 mL per sample) and stored at −80°C prior to analysis. All samples were collected within 20 min of being removed from the 37°C incubator. For LC-MS, 200 μL aliquots were lyophilized. One hundred microliter aliquots were combined to prepare a pooled QC sample from which 200 μL aliquots were lyophilized. Samples were analyzed in a random order applying UHPLC-MS as described below.

#### Chemicals

Acetonitrile, methanol, isopropanol, and water (HPLC grade) were purchased from Fisher Scientific (Loughborough, UK). Formic acid and acetic acid (≥98.0% purity) were purchased from VWR International (Lutterworth, UK), and ammonium formate and ammonium acetate (≥98.0% purity) were purchased from Sigma-Aldrich (Poole, UK).

#### Sample analysis

Each biological sample, QC sample, and blank sample was analyzed applying two complementary UHPLC-MS assays: a HILIC assay in positive and negative ion modes to study water-soluble metabolites. The samples were maintained at 4°C and analyzed applying two Ultra Performance Liquid Chromatography-Mass Spectrometry (UPLC-MS) methods using a Vanquish Liquid Chromatography System UPLC+ (Thermo Fisher Scientific, MA, USA) coupled with a heated electrospray Q Exactive Plus mass spectrometer (Thermo Fisher Scientific, MA, USA). Ten pooled QC samples were analyzed at the start of the analytical batch to condition the analytical system. Pooled QC samples were then analyzed after every sixth biological sample and twice at the end of the analytical batch. The process (extraction) blank samples were analyzed as injection six and as the last injection of the batch. All analysis order of biological samples was randomized applying the RAND() function in Microsoft Excel.

##### HILIC assay

Polar extracts were analyzed on an Accucore-150-Amide-HILIC column (100 × 2.1 mm, 2.6 μm, Thermo Fisher Scientific, MA, USA). For positive ion mode analysis, mobile phase A consisted of 10 mM ammonium formate and 0.1% formic acid in 95% acetonitrile/water and mobile phase B consisted of 10 mM ammonium formate and 0.1% formic acid in 50% acetonitrile/water. For negative ion mode analysis, mobile phase A consisted of 10 mM ammonium acetate and 0.1% acetic acid in 95% acetonitrile/water and mobile phase B consisted of 10 mM ammonium formate and 0.1% acetic acid in 50% acetonitrile/water. For both positive and negative ion mode, the flow rate was set for 0.50 mL·min-1 with the following gradient: t = 0.0, 1% B; t = 2.1, 1% B; t = 4.1, 15% B; t = 7.1, 50% B; t = 10.1, 95% B; t = 11.0, 95% B; t = 11.5, 1% B; t = 15.0, 1% B, all changes were linear with curve = 5. The column temperature was set to 35°C and the injection volume was 2 μL. Data were acquired in positive and negative ionization modes separately within the mass range of 70–1,050 m/z at resolution 70,000 (FWHM at m/z 200). Ion source parameters were set as follows: Sheath gas = 55 arbitrary units, Aux gas = 14 arbitrary units, Sweep gas = 4 arbitrary units, Spray voltage = 3.2 kV (positive ion) / 2.7 kV (negative ion), Capillary temp. = 380°C, Aux gas heater temp. = 440°C. Data dependent MS2 in “Discovery mode” was used for the MS/MS spectra acquisition applying a pooled QC sample for each sample type using the following settings: resolution = 17,500 (FWHM at m/z 200); Isolation width = 3.0 m/z; stepped collision energies (stepped CE) = 20, 40, 100 [positive ion mode] / 40, 60, 130 [negative ion mode]. Spectra were acquired in five different mass ranges with each range acquired for a separate QC sample injection (QC samples 6–10): 70–210 m/z; 200–310 m/z; 300–410 m/z; 400–510 m/z; 500–1,050 m/z. A Thermo ExactiveTune 2.8 SP1 build 2806 was used as instrument control software in both cases, and data were acquired in profile mode.

### Raw data processing and univariate analysis

Vendor format raw data files (.RAW) were converted to the mzML file format using ProteoWizard software. Deconvolution was performed by the XCMS R package (version 3.12 running in R version 4.0.5). XCMS was operated applying min peak width (6 s); max peak width (30 s); ppm (14); mzdiff (0.002); bw (0.25); mzwid (0.01); minfrac (0.2). A data matrix of peak areas for metabolite features (m/z-retention time pairs) vs samples was constructed for each assay. Data for the first eight QC samples were removed from the data set prior to further processing and analysis. Each data matrix was filtered as follows: any feature whose median intensity in the biological samples was <20 × its median intensity of the extraction blank samples was removed; any feature present in <70% of the QC samples was removed; features with RSD ≥ 30% across the pooled QC samples (QC9 to last QC sample analyzed) were removed. Putative metabolite annotation applying MS1 data was performed using the Python package BEAMSpy (https://github.com/computational-metabolomics/beamspy). The parameters applied were maximum retention time = 2 s; grouping method = Spearman Rank (Coefficient threshold = 0.5, *P* < 0.05) and mass error ±5 ppm. Metabolite annotation based on the Human Metabolome Database, KEGG (human), and LIPIDMAPS with a mass tolerance of ±5 ppm was applied. Statistical and pathway enrichment analysis applied to the metabolomics data sets was performed in MetaboAnalyst v.5.0. For statistical analysis, data were normalized to total sample response and log10 transformed. Statistical analysis applied one-way ANOVA (*P* < 0.05).

### *C. albicans* growth assays, chitin exposure, and hyphal growth measurements

In some experiments, *C. albicans* was grown in the presence of vancomycin for 24 h. *C. albicans* was seeded into 96-well plates at 5,000 yeast/well, in the presence or absence of vancomycin (200–100 μg/mL). Yeast growth was monitored by serial readings at OD_600_ using a FLUOstar Omega microplate reader. data were averaged across three wells per growth condition. For chitin staining, yeast cells grown in YPD media, or YPD supplemented with 20, 50, or 100 µg/mL vancomycin, were washed and stained with 10 μg/mL wheat germ agglutinin (WGA; Invitrogen) on ice for 30 min, followed by 3.5 μg/mL of calcofluor white (CFW; Sigma-Aldrich) and incubating on ice for 10–15 min. Labeled yeast cells were analyzed using a BD Fortessa flow cytometer, as above. Total (CFW) or exposed (WGA) chitin staining was calculated using the median fluorescence intensity of the cells. For hyphal measurements, length was measured using ImageJ software.

### Statistics

Statistical analyses were performed using GraphPad Prism 9.0 software. Details of individual tests are included in the figure legends. In general, data were tested for normal distribution by Kolmogorov-Smirnov normality test and analyzed accordingly by unpaired two-tailed *t*-test or Mann-Whitney *U*-test. In cases where multiple data sets were analyzed, two-way ANOVA was used with Bonferroni correction. In all cases, *P*-values <0.05 were considered significant.

## SUPPLEMENTAL MATERIAL

Supplemental Figures mbio.00580-26-s0001.pdf Figures S1 to S4.

Table S1 mbio.00580-26-s0002.csv Raw gene counts from RNA sequencing of uninfected and infected macrophages.

Table S2 mbio.00580-26-s0003.xlsx Differentially expressed genes after infection in control and vancomycin-treated macrophages.

Table S3 mbio.00580-26-s0004.xlsx Shared and unique genes upregulated during infection by control and vancomycin-treated macrophages.

ASM does not own the copyrights to Supplemental Material that may be linked to, or accessed through, an article. The authors have granted ASM a non-exclusive, world-wide license to publish the Supplemental Material files. Please contact the corresponding author directly for reuse.
