# Supplementary material for: Vancomycin disrupts mitochondrial morphology and function and impairs macrophage fungal killing
Source: mBio. 2026 Apr 29;17(6):e00580-26. doi: 10.1128/mbio.00580-26 (PMC13251413; doi:10.1128/mbio.00580-26)
Supplement: Supplemental Figures — Figures S1 to S4. [file mbio.00580-26-s0001.pdf]

Supplemental Figures

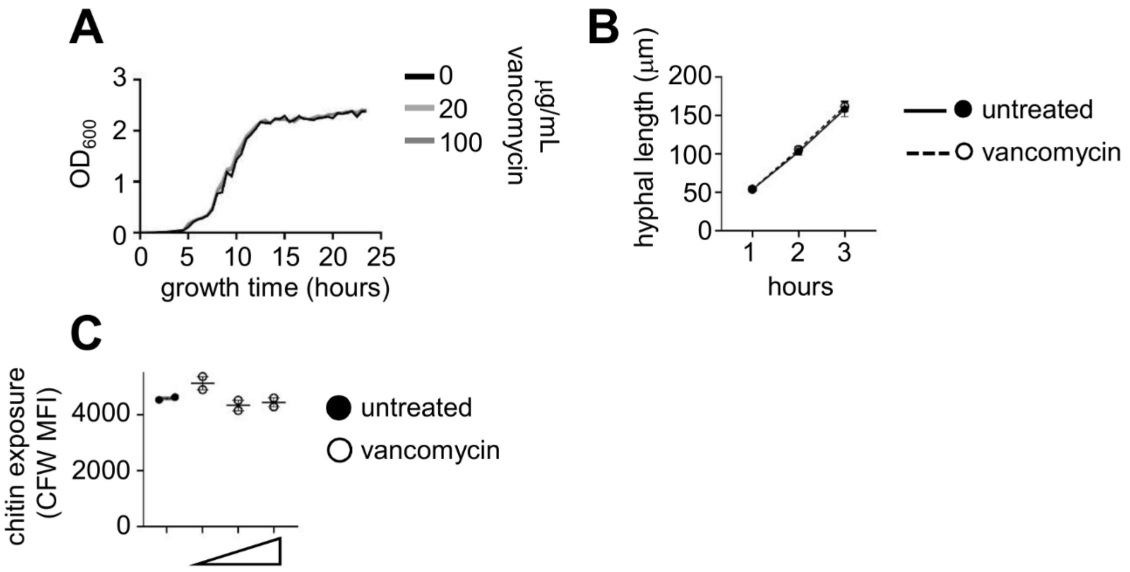

Figure S1: (A) *C. albicans* yeast were grown in the presence or absence of vancomycin and growth measured via absorbance at 600nm for 24 hours. Data shown is from a single representative experiment that was repeated 3 times. (B) The average hyphal length at indicated time points following growth in hyphal induction medium. Hyphal lengths were measured from at least 50 cells per time point per condition. (C) Surface chitin exposure as determined by mean fluorescence intensity (MFI) of calcofluor white (CFW) staining and analysis by flow cytometry. Points represent average values from individual experiments. The 3 vancomycin concentrations tested are 20, 50 and 100 μg/mL. Yeast were exposed to vancomycin for 2 hours prior to staining and analysis.

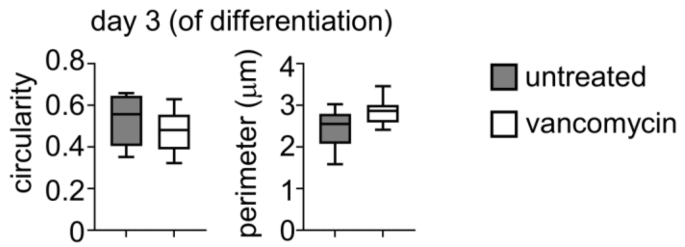

Figure S2: Circularity and perimeter measurements of mitochondria in untreated or vancomycin-treated macrophages at day 3 during differentiation. Mitochondrial measurements were taken from n=10 macrophages per condition. Box-and-whisker plots show min-to-max.

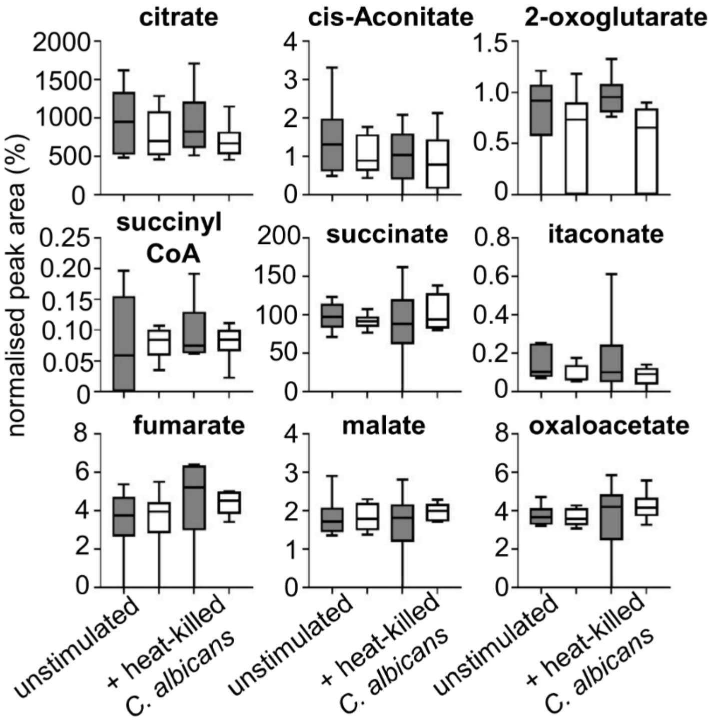

Figure S3: LC-MS data measuring levels of indicated citric acid cycle intermediates in untreated (filled bars) or vancomycin-treated (white bars) macrophages unstimulated or treated with heat-killed *C. albicans* for 2 hours. Data pooled from 6 individual experiments (using macrophages generated from individual animals). Box-and-whisker plots shown as min-to-max.

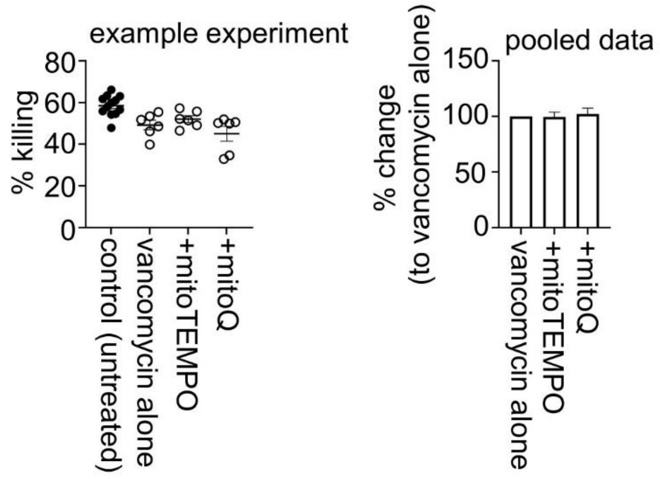

Figure S4: Inhibition of mitochondrial ROS does not affect fungal killing by vancomycin-treated macrophages. Untreated and vancomycin-treated macrophages were challenged with *C. albicans* for 2 hours and fungal viability measured using Alamar Blue (see Methods). Vancomycin-treated macrophages were additionally treated with mitochondrial ROS inhibitors, mitoQ and mitoTEMPO. Graph of the left shows data from an example experiment, where each dot represents a technical replicate (i.e. well, n=6-12 wells per condition). Graph on the right shows pooled data (n=3 sets of macrophages generated from individual mice, analysed on different days), normalised to vancomycin-treated cells with no additional drugs. Bar graphs shown as mean +/- SEM.
